# Management of Women with Surgically Staged 1 Uterine Papillary Serous Cancer

**DOI:** 10.5402/2011/146264

**Published:** 2011-09-11

**Authors:** Laurie Elit, Andre LaRoche, Lauren Smith, John Mazurka, Francois Moens, Jan Hauspy, Waldo Jimenez

**Affiliations:** ^1^Department of Obstetrics and Gynecology, McMaster University, Hamilton, ON, Canada L8N3Z5; ^2^Department of Gynecologic Oncology, St. Augustinus Hospital, L8V 5C2 Antwerp, Belgium

## Abstract

*Objective*. To review the management and outcomes of women with surgically staged 1 UPSC. *Methods*. We report on a case series from 2008–2010 from Hamilton Canada. We summarize the data from a literature search on surgically staged 1 UPSC. *Results*. There is a group women with Stage 1A UPSC with no residual disease at time of surgery who do not require adjuvant therapy. Vault recurrences appear to be lower in women who received adjuvant vault radiation. Chemotherapy appears to confer longer survival for those women with Stage 1B or 1C disease compared of those observed or who had radiation alone. *Conclusion*. Adjuvant therapy appears to confer benefit in certain groups of women with stage 1 UPSC. A randomized controlled study would clarify the degree of benefit.

## 1. Background

Karpas first described an endometrial cancer with psammomatous bodies in 1963 [1]. Uterine papillary serous cancer (UPSC) was described as a distinct entity by Lauchlan in 1981 [2]. Hendricks noticed that although UPSC represented 4% of all uterine cancers; it accounted for 50% of the uterine cancer recurrences [2–10]. In fact, early stage UPSC accounted for 25% of deaths from uterine cancer [2–4, 11–23]. The poor prognosis of UPSC was explained in part due to the unrecognized presence of metastatic disease at initial presentation. Goff showed that 72% of women with clinical Stage 1 UPSC had extrauterine disease at the time of surgery in comparison to a rate of 25% in women with endometrioid cancer. In Goff's study, the retroperitoneal nodes were positive in 36% of women without invasive disease, 50% with inner half myometrial involvement and 40% with outer half myometrial involvement. Peritoneal disease was seen in 43%, 37%, and 35%, respectively [24–29]. More recently, it was noted that even surgically staged patients, who had Stage 1 UPSC, had a propensity to develop recurrent disease [30, 31]. If women with surgically staged early disease have poor outcomes, the next questions are whether there is any adjuvant treatment that could either improve or prolong duration of survival. Our objective in this paper was to document the 2-year survival and recurrence rate of women with surgically staged 1 UPSC treated with adjuvant chemotherapy and/or radiation therapy. As no one centre has many cases, we planned to review the literature to see if any themes arose.

## 2. Methods

### 2.1. Case Series

In 2009, our disease site team reviewed the literature and subsequently changed out treatment policy for women with Stage 1 UPSC. We moved from observation only to offering the sandwich technique of 3 cycles of carboplatin followed by brachytherapy and then 3 further cycles of carboplatin. Women with UPSC were identified by three strategies: the pathology database at Hamilton Health Sciences Centre (this includes all surgical cases done on site and all pathology consults from the local health integrated network), the cancer centre database (this includes all new patient referrals to the cancer centre), and a review of all weekly gynaecologic oncology tumour board meeting minutes from 2008 to present. The search terms included serous uterine cancer and UPSC. The charts of all cases were reviewed. FIGO staging was according to the 1988 classification. Only women who were Stage 1 by surgically staging with washings, hysterectomy, bilateral salpingoophorectomy, omentectomy, and retroperitoneal node dissection were included.

Disease characteristics included depth of invasion, amount of serous component and lymphovascular space involvement. Amount of serous component was defined as pure if the specimen contained at least 90% serous cancer and mixed if it did not.

Management included chemotherapy with platinum taxane usually with carboplatin AUC 5 based on 24 collection of urinary creatinine clearance and a 3-hour infusion of paclitaxel 175 mg/m^2^ q3 weeks. Radiation involved either vault brachytherapy to a dose of 1050 cGy and/or whole pelvis radiation to 4500 cGy. 

### 2.2. Literature Review

Search strategy included MEDLINE (1980–2011), EMBASE (1980–2011) and the Cochrane Library databases for systematic reviews and clinical trials. Reference lists of papers and review articles were scanned for additional citations. Abstracts from 1997 to 2011 annual meetings of the American Society of Clinical Oncology (ASCO) were also searched. Canadian Medical Associate (CMA) Infobase (https://mdm.ca/cpgsnew/cpgs/search/english/help/help-glossary.html), the National Guidelines Clearinghouse (http://www.guideline.gov/), and other web sites were searched for existing evidence-based practice guidelines. Search terms related to study design, used to search the MEDLINE and EMBASE databases, included case series, cohort studies, clinical trials meta-analysis and systematic review. The following test words and medical subject headings (MeSH) were used to identify the literature regarding adjuvant therapy in women with serous cancer of the uterine: uterine papillary serous carcinoma, drug therapy, chemotherapy, and radiation therapy. To be included in this paper, patient could have pure or mixed UPSC, all women were surgically staged Stage 1 disease, or the author indicated that the rate of surgically staging was 80% or higher and the study is about this population or we are able to pull out specific patients from the details in the patients that were surgically staged and their adjuvant management and outcomes information were clearly defined. 

## 3. Results

### 3.1. Case Series

We report on 26 Stage 1 UPSC cases who underwent surgery. Five are excluded as they did not have retroperitoneal node dissection. There were 6 women with Stage 1A disease. All patients had residual tumour in the uterus at the final pathology. There were 10 women with Stage 1B and 5 women with Stage 1C diseases. 

In those with Stage 1A disease, only 1 patient received the recommended treatment, 2 only received chemotherapy and, 3 received no treatment. In those with Stage 1B disease, 5 received carboplatin and paclitaxel plus brachytherapy, 1 received carboplatin and paclitaxel, alone and 4 received no treatment. In those with Stage 1C disease, 3 received carboplatin with brachytherapy, 1 received carboplatin with pelvic radiation, 1 received carboplatin alone, and 1 had no treatment. There were 2/9 cases of treatment delay due to neutropenia in the group with the sandwich technique. There were 2/4 cases of treatment delay due to thrombocytopenia in the chemotherapy-only group. Our disease site team had a 50% compliance with the guideline. 

All patients are alive at a median of 16-month followup (range 4–40, mean 16.4 mos). There were 2 recurrences at 12 and 13 months of followup. Both were vaginal recurrences in patients with Stage 1C disease. In both cases, the patients had not received upfront radiation therapy.

### 3.2. Literature Search Results

 There was one prospective Phase 2 study addressing feasibility and toxicity of the sandwich technique of 3 cycles of chemotherapy then radiation and 3 further cycles of chemotherapy [32]. 16 retrospective studies reported survival and recurrence outcomes for women with surgically staged Stage1 UPSC. 9 studies focused on Stage 1 disease [22, 29, 33–41]. The other 8 studies discussed all stages [28, 32, 42–47] but with enough information so that the Stage 1 patients could be identified and their outcomes were available.

### 3.3. Management

Most of the women in these studies began their management with surgical staging. Surgical staging involved hysterectomy, bilateral salpingoophorectomy, as well as retroperitoneal node dissection, omentectomy, and washings with or without peritoneal biopsies. The importance of surgical staging has been identified by several authors. Unlike endometrioid uterine cancer, in UPSC, metastatic disease cannot be reliably predicted based on uterine factors as women with apparent early disease (i.e., involving a polyp) may have already widespread intra-abdominal metastases. Thomas et al. [41] showed that 30% of women without clinical evidence of extrauterine disease at surgery were upstaged by the routine biopsies. Thomas et al. [41] in his series noted a clear shift in their practice in 1995 to surgically staging women with UPSC patients (i.e., at least a pelvic node dissection). 

In 12 studies, women received just adjuvant chemotherapy. In 13 studies, the women received some form of adjuvant radiation: (a) vault radiation, (b) pelvic radiation, (c) whole abdominal pelvic radiation (WART), or (d) a combination of radiation techniques. In 8 studies, women received a combination of chemotherapy and one or more radiation techniques. 

There is a clear bias in the retrospective studies to provide women with deeper myometrial involvement with adjuvant therapy. For example, in Elit et al. [35], 86% of women with Stage 1A disease received no adjuvant therapy, whereas 88% with Stage 1C disease received adjuvant therapy. Conclusions about adjuvant therapy may be difficult to draw based on this bias. However, reviewing case series can help us identify some themes that would define future directions. For example, do all patients require adjuvant therapy? If there is a specific group of women such as those with no myometrial involvement (i.e., Stage 1A) that has excellent outcomes without adjuvant therapy, this would be important to know. If, however, all patients had poor outcomes, an argument could be made not to do extensive surgical staging and just provide adjuvant therapy. 

Table 1 shows the surgical staging and histology information of the surgically staged patients included in this study.  Table 2 shows the risk of recurrent disease. Certainly, there appears to be a gradient of increase risk of recurrent disease as myometrial invasion increases from Stage 1A to 1C. The numbers are low, but serous versus mixed tumours and presence or absence of lymphovascular invasion do not appear to predict recurrence. 

Tables 3 and 4 shows the information on different patterns of adjuvant therapy. Certainly, the only group with significant number are the women who had surgical staging and were observed.  Table 5 defines the recurrence rate by adjuvant treatment and stage.

Looking at the observation group alone, there is a substantial difference in recurrence rates between those with Stage 1A and the other Stages 1B-1C. 

Looking at the individual studies, some authors have advocated *observation* only after surgical staging and disease truly limited to the uterus [22, 28, 29, 33, 35–44, 46, 47]. Others like Kelly et al. [40] showed that the greater the myometrial invasion, the more likely a recurrence is seen with observation alone (43% Stage 1A recurred, 77% Stage 1B recurred, 80% Stage 1C recurred). There does appear to be a gradient in risk of recurrence by Stages 1A,B,C. When focusing on Stage 1A UPSC, some authors showed no risk of relapse [22, 32] or a low risk of relapse up to 12% [27, 43, 48–50]. Works by Hui et al. [51] and Kelly et al. [40] have shown no recurrences in women observed with Stage 1A disease without residual UPSC, this is in contrast to a 43% recurrence rate for Stage 1A patients with residual uterine disease in the hysterectomy specimen who were observed. Thus, they suggest no adjuvant therapy for just those women with no residual uterine disease at time of definitive surgical staging. 

Looking at whether radiation improved survival over and above observation alone, the only group that appeared to benefit was the Stage 1B population. The individual studies addressed various forms of radiation therapy. Vault radiation has been used to decrease vaginal relapse. Pelvic radiation has been used to decrease locoregional recurrence. Whole abdominal pelvic radiation therapy has been used to sterilize the whole abdominal cavity (WART). 

Huh et al. [29] and Kelly et al. [40] showed that if patients with surgical Stage 1 disease were not treated with *pelvic or vault radiation,* there was a 10–19% chance of recurring at the vaginal vault. The risk of vault recurrence appears to be related to depth of myometrial invasion. There was a 29% rate of vault recurrence in women with Stage 1B and 1C diseases in absence of adjuvant RT [41]. Vault radiation appears to decrease recurrences as compared to no adjuvant therapy. Kelly reported a vaginal recurrence rate of 19% (6/13) in Stage 1 UPSC in absence of adjuvant RT versus none in the 43 women who received RT (VB = 38, pelvic RT = 5). Use of *WART* in UPSC was first reported by Frank et al. [52] and others [53, 54]. The earlier studies suggested a benefit from WART in patients with Stage 1–3 disease. It was difficult to comment on outcomes of WART in Stage 1 disease given the information available. The results from adjuvant radiation alone still result in a high distant recurrence rate. 

Looking at chemotherapy alone, again the numbers per strata are small, but there is a trend to markedly lower recurrences across the Stages 1A to 1C compared to observation alone or radiation. Several *chemotherapy* agents have been used alone or in combination for this disease. The most common agents used either alone or in combination include platinum, taxane, doxorubicin, and cyclophosphamide [8, 30, 55, 56]. In the individual studies, Fader et al. [37, 38] showed that those 89 patients who had chemotherapy had a superior OS and PFS (81.5% and 87.6%) compared to those 33 women observed (64.7% and 70.2%) and the 20 women treated with radiation (64.1% and 59.5%). On multivariate analysis, only chemotherapy significantly and favourably impacted upon recurrence rates and PFS (*P* = 0.006). Age (*P* = 0.05), stage (*P* = 0.05), and chemotherapy (*P* = 0.02) were associated with overall survival [38]. Kelly et al. [40] showed that in those Stage 1A patients given chemotherapy there were no recurrence compared to 43% recurrence in those not given chemo. In Stage 1B patients treated with chemotherapy, there were no recurrences compared to 77% in those not treated with chemotherapy. In Stage 1C patients treated with chemotherapy, there were no recurrences compared to 80% in those not treated with chemotherapy. Overall, you had a 100% chance at survival with chemotherapy compared to 46% without chemotherapy. Dietrich et al. [34] showed in a multi-institutional retrospective study that all 21 surgically staged 1 patients treated with 3–6 cycles of carboplatin taxane were alive and well at a minimum of 8 mos. One had developed a vaginal recurrence that was managed with radiation. In 8 patients treated with other platinum single agent or combinations, 10-year survival was only 85% with 2 recurrences, 1 distant disease who died at 24 mos and 1 vaginal recurrence still alive after retreatment with chemotherapy and radiation. Huh et al. [29] showed no recurrences in the 7 patients treated with platinum-based chemotherapy. Fader et al. [38] showed that those with chemotherapy had a reduction in recurrences (11.2%) compared to those who did not have chemotherapy (28.3%, *P* = 0.013). PFS for CT-treated patients was statistically significantly higher than for those who did not receive CT (*P* = 0.013). CT with or without RT was associated with improved PFS compared with treatment with RT also or observation (*P* = 0.027). This was most pronounced for Stage 1B patients. Overall survival did not reach statistical significance.

The problem with chemotherapy alone was a concern for the high rate of recurrences especially at the vaginal apex. Looking at that chemotherapy and radiation data, the cell sizes are larger than the chemotherapy alone data. However, the rate of recurrences by substage look similar to the chemotherapy alone data and much better than the observation alone data. The individual studies on *sequential or concurrent chemoradiation* also suggest benefit [10, 40, 57]. Havrilesky et al. [39] (Table 6) showed that the 3 yr OS and PFS for chemo (92%, 76%) or chemo with radiation (89%, 77%) were superior to observation (80%, 78%) or radiation alone (63%, 44%). Fader et al. [38] showed that chemoradiation provided a superior recurrence outcome than radiation alone (*P* = 0.027). Turner showed a superior 5 yr OS in the women receiving chemotherapy and vault RT (94%) compared to those treated with WART + vault RT (65%). Rosenburg et al. [8] offered 8 Stage 1 patients chemotherapy and radiation and there were no recurrences at 32 mos. Bancher-Todesca et al. [42] treated 4 Stage 1 patients with platinum and pelvic radiation and they are all alive at 39 mos. Low et al. [45] reported on 9 Stage 1 patients being treated with 4 cycles of platinum followed by pelvic radiation and in 5 cancer vault brachytherapy. There was 1 recurrence. 

A novel chemoradiation approach dubbed the “sandwich technique” was evaluated by Fields et al. [32]. He conducted a prospective pilot study to assess the feasibility of 3 courses of chemotherapy, then pelvic radiation therapy followed by 3 further cycles of chemotherapy. Patients could have had any stage of UPSC. The treatment was tolerable with 97% of patients completing the intended treatment. 42% for the cycles were associated with grade 3/4 neutropenia and only 3% needed a one-week treatment delay. There were no cases of febrile morbidity.

## 4. Discussion

We have pooled the data across various-case series in surgically staged women with Stage 1 UPSC. We show that the recurrence rates of those observed is significantly different in those with Stages 1B and 1C compared to those with Stage 1A disease. We show that recurrence rates are improved especially in those with Stage 1B and 1C diseases who receive adjuvant therapy including chemotherapy. To minimize vault recurrence, the addition of at least vault brachytherapy has been recommended. Our small case series reinforces this issue. However, the strata sizes in this study are too small to make this conclusion strongly. Although there is agreement to offer adjuvant treatment to women with Stage 1B and 1C diseases, the evidence is not as compelling as for the adjuvant treatment of those with Stage 1A disease. There appears to be a subgroup of Stage 1A patients without residual disease in the hysterectomy specimen which has a low chance of recurrence in whom observation seems appropriate. 

There are significant limitations of this paper. We use the 1988 FIGO staging system in order to compare studies using the same staging classification. Given the new 1998 FIGO staging system, the old Stages 1A and 1B will be collapsed into Stage 1A. The old Stage 2A will now be absorbed within Stage 1. The subtleties of management and outcomes using the old staging system will be lost when categorizing patients with new system. The plethora of small case series exist because of the relative rarity of surgically staged 1 UPSC cases. Some of the issues that exist in trying to compare these studies include that many studies involved single versus multiple centres. Data collection in multicentre studies is more likely to be stringent as a data dictionary would have been used to define and collect similar data. Most of the studies are retrospective with the exception of prospective study by Fields. Thus, there is a strong risk of bias for treatment as we saw for the women with Stages 1B and 1C. In earlier reports, management results grouped UPSC with other high-grade cancers like clear cell; thus, it was difficult to discern the outcome of the UPSC cases and likely those cases were excluded. Many earlier studies reported on all stages of UPSC or a combination of Stages 1 and 2 versus focusing in on just Stage 1. Again, the information from these studies was likely excluded. The quality of the surgical staging varied across studies, that is, retroperitoneal node dissection only versus addition of omentectomy and peritoneal biopsies. Many earlier studies report their “experience with UPSC” and describe several adjuvant treatment strategies with small number of cases in each strata. Bias in choosing a management strategy is clearly an issue here. The radiation techniques varied across studies. Follow-up time in some studies was short, that is, 3 years; however, most recurrences were noted within the first 2 years after treatment, thus the impact of this short follow-up is not as concerning as may be the case in other disease types.

A randomized study provides the best opportunity for defining management. However, given the limitations listed about a randomized study in this rare entity is unlikely. This study of the current literature does provide themes around improvements for care in women with Stage 1 UPSC. There appears to be a role for chemotherapy in preventing the high rate of distant disease. There appears to be a role for some form of radiation, either vault brachytherapy or whole pelvic radiation to decrease the high risk of vault relapses.

## Figures and Tables

**Table 1:** Characteristics of the Stage 1 UPSC studies included in this paper.

Author	Publication year	Single- or multi-institutional	Time period	Number of Stage 1 patients	Median FUP mos
Bancher-Todesca et al. [42]	1998	Multi	1988–1995	3	*36*
Bristow et al. [28]	2001	Single	1989–1998	11	*46*
Carcangiu et al. [33]	1997	Multi	1987–1995	13	*38*
Dietrich et al. [34]	2005	Multi	1990–2003	29	*31*
Elit et al. [35]	2004	Multi	1985–2001	43	*40*
Fader et al. [36–38]	2009	Multi	1993–2006	142	*37*
Fields et al. [32]	2008	Single	1999–2004	16	*50*
Gallion et al. [22]	1989	Single	1973–1987	11	*NR*
Gehrig [44]	2001	Single	1990–2000	6	*24*
Grice et al. [43]	1998	Single	1982–1993	12	*43*
Huh et al. [29]	2003	Multi	1987–2000	60	*30*
Havrilesky et al. [39]	2007	Multi	1976–2006	83	*NR*
Kelly et al. [40]	2005	Single	1987–2004	74	*NR*
Low et al. [45]	2005	Single	1994–2003	9	*28*
Piura et al. [46]	1998	Multi	1991–1997	7	*35*
Slomovitz et al. [47]	2003	Single	1989–2002	52	*NR*
Thomas et al. [41]	2007	Single	1982–2005	42	*39 8*
Elit et al.*	2011	Single	2008–2011	21	*16*

TOTAL				**613**	

*Data from Elit are not added to summary statistics as median followup is less than 24 months.

**Table 2:** Disease characteristics of the patients in these studies.

Author	Stage (numbers)				Serous	Mixed	LVI (percent of cases)		
	1A	1B	1C	Total			+	−	Unknown
Bancher-Todesca et al. [42]	1	1	1	3	—	—	—	—	—
Bristow et al. [28]	4	7	—	10	—	—	—	—	—
Carcangiu et al. [33]	13	—	—	13	—	—	—	—	—
Dietrich et al. [34]	7	17	5	29	16	5	—	—	—
Elit et al. [35]	22	13	8	43	36 83%	7 17%	—	—	—
Fader et al. [36–38]	51	65	26	142	—	—	—	—	—
Fields et al. [32]	2	12	2	16	—	—	—	—	—
Gallion et al. [22]	3	5	3	11	—	—	7 64%	4 36%	—
Gehrig [44]	6	—	—	6	—	—	5 83%	1 17%	—
Grice et al. [43]	4	4	4	12	—	—	—	—	—
Huh et al. [29]	19	26	15	60	51 85%	10 15%	—	—	—
Havrilesky et al. [39]	32	34	16	82	—	—	18 22%	54 65%	11 13%
Kelly et al. [40]	33	29	12	74	47 64%	27 36%	—	—	—
Low et al. [45]	1	6	2	9	—	—	—	—	—
Piura et al. [46]	1	3	3	7	—	—	—	—	—
Slomovitz et al. [47]	19	26	7	52	27 56%	23 44%	12 23%	40 77%	—
Thomas et al. [41]	15	21	6	42	—	—	—	—	—
Elit et al.*	6	10	5	21	19 90%	2 10%	3 14%	18 86%	—

TOTAL	233	269	110	611	177/258 69%	72/258 28%	42/151 28%	99/151 66%	11/82 13%

*Data from Elit are not added to summary statistics as median followup is less than 24 months.

**Table 3:** Disease recurrences by disease characteristic.

Author	Stage			Serous	Mixed	LVI		
	1A	1B	1C			+	−	Unknown
Bancher-Todesca et al. [42]	0/1	0/1	0/1	—	—	—	—	—
Bristow et al. [28]	1/ 4	1/7	—	—	—	—	—	—
Carcangiu et al. [33]	2/13	—	—	—	—	—	—	—
Dietrich et al. [34]	0/7	2/17	1/5	3/16	0/5	—	—	—
Fader et al. [36–38]	6/51	11/65	8/26	—	—	—	—	—
Fields et al. [32]	0	3/12	2/2	—	—	—	—	—
Gallion et al. [22]	0/3	4/5	1/3	—	—	5/7	0	—
Gehrig [44]	2/6	—	—	—	—	—	—	—
Grice et al. [43]	0/4	0/4	2/4	—	—	—	—	—
Havrilesky et al. [39]	—	—	—	—	—	18	53	11
Kelly et al. [40]	6/33	6/29	8/12	—	—	—	—	—
Low et al. [45]	0/1	1/6	0/2	—	—	—	—	—
Piura et al. [46]	0/1	0/3	1DOC/3	—	—	—	—	—
Slomovitz et al. [47]	4/19	—	—	—	—	—	—	—
Elit et al.*	0/6	0/10	2/5	2/19	0/2	1/3	1/18	
	21/143 (15%)	31/149 (21%)	22/56 (39%)	19%	0	22%	65%	13%

*Data from Elit are not added to summary statistics as median followup is less than 24 months.

**Table 4:** Description of management.

Author	Observe	RT					CT	CT	3CT-RT-3CT
		Vault	Pelvic	Pelvic + Brachy	WART	Other		Brachy	
Bancher-Todesca et al. [42]	2	—	—	1	—	—	—	—	—
Bristow et al. [28]	10	1	—	—	—	—	—	—	—
Carcangiu et al. [33]	1	—	—	—	2	—	—	10	—
Dietrich et al. [34]	—	—	—	—	—	—	28	—	—
Elit et al. [35]	27	—	4	—	6	—	6	—	—
Fader et al. [36–38]	33	—	—	—	—	20	—	89 CT with or without RT	—
Fields et al. [32]	—	—	—	—	—	—	—	—	16
Gallion et al. [22]	5	—	—	6	—	—	—	—	—
Gehrig [44]	6	—	—	—	—	—	—	—	—
Grice et al. [43]	5	—	4	2	1	—	—	—	—
Huh et al. [29]	40	4	—	5	3	—	7	—	—
Havrilesky et al. [39]	47	7	10	4	9	—	17	—	—
Kelly et al. [40]	11	3	—	—	1	—	21	8	—
Low et al. [45]	—	—	—	—	—	—	—	9	—
Piura et al. [46]	3	—	—	3	—	—	1	3	—
Slomovitz et al. [47]	27	6	2	—	3	—	5	1	—
Thomas et al. [41]	17	9	6	—	3	—	2	5	—
Elit et al.*	8	—	—	—	—	—	4	—	9
	234	30	26	21	28	20	87	125	16

*Data from Elit are not added to summary statistics as median followup is less than 24 months.

**Table 5:** Recurrence rate by adjuvant treatment and stage of disease.

Author	Observation			Radiation			Chemotherapy			CT+RT		
	1A	1B	1C	1A	1B	1C	1A	1B	1C	1A	1B	1C
Bancher-Todesca et al. [42]	0/1		0/1		0/1							

Bristow et al. [28]	1/ 4				1/1							

Carcangiu et al. [33]	0/1			1/ 2						1/10		

Dietrich et al. [34]							0/7	2/17	1/5			

Elit et al. [35]	2/19	3/7	0/1		0/5	3/5						

Fader et al. [36–38]	3/19	4/9	3/5	1/5	2/9	2/6				2/27	5/47	3/15

Fields et al. [32]										0/2	3/12	2/2

Gallion et al. [22]	0/3	0/1	1/1	0/0	4/4	1/2						

Gehrig [44]	2/6											

Huh et al. [29]				1 /3	0/6	1 /3	0/2	0/3	0/2	0/1		

Low et al. [45]										0/1	1 /6	0/2

Piura et al. [46]		1 /2	0/1 1DOC		0/3 1DOC				1/1	0/1		0/2

Thomas et al. [41]	0/11			0/3			0/1					

Elit et al.*	0/3	0/4	1/1				0/2	0/1	1/1	0/1	0/5	0/3

Total	8/64	8/19	4/9	3/13	1/29	7/16	0/10	2/20	2/8	3/42	9/65	5/21

Percent	12.5%	42%	44%	23%	3%	43.7%	0	10%	25%	7%	14%	25%

Stage 1A	12.5%			23%			0			7%		

Stage 1B		42%			3%			10%			14%	

Stage 1C			44%			43.7%			25%			25%

*Data from Elit are not added to summary statistics as median followup is less than 24 months.

**Table 6:** Outcomes report by study.

Author	5 yr OS	5 yr PFS	Other	1A	1B	1C
Bristow et al. [28]	82%					

Carcangiu et al. [33]	83%					

Dietrich et al. [34]		85.7% platinum + cyclo or platinum alone100% for carbo + taxol				

Fields et al. [32]			3 yr OS 75% 3 yr DFS 69%			

Grice et al. [43]	84%		48 mos′			

Huh et al. [29]	72%	70%				

Havrilesky et al. [39]			Rads only 3 yr OS 63% 3 yr PFS 44%	3 yr OS 95%, PFS 85%	3 yr OS 69%, PFS 59%	3 yr OS 79%, PFS 60%

Kelly et al. [40]				51 mos′	37 mos′	44 mos′

Low et al. [45]	73%					

Piura et al. [46]			83.3% 3 yr for 10 patients only 7 surgically staged	34 mos′	46 mos′	35 mos′

Slomovitz et al. [47]	63%			5 yr OS 81.5%	5 yr OS 58.6%	5 yr OS 34.3%

Thomas et al. [41]	85%	78%		5 yr OS 100%	5 yr OS 89%	5 yr OS 60%

5 yr OS.

DFS: disease free survival.

′: Median survival.
